# Serum levels of Asprosin in patients diagnosed with coronary artery disease (CAD): a case-control study

**DOI:** 10.1186/s12944-021-01514-9

**Published:** 2021-08-21

**Authors:** Nariman Moradi, Fatima Zahraa Fouani, Akram Vatannejad, Abbas Bakhti Arani, Soraya Shahrzad, Reza Fadaei

**Affiliations:** 1 Cellular and Molecular Research Center, Research Institute for Health Development, Kurdistan University of Medical Sciences, Sanandaj, Iran; 2 Department of Cellular and Molecular Nutrition, School of Nutritional Sciences and Dietetics, Tehran University of Medical Sciences, Tehran, Iran; 3 Department of Comparative Biosciences, Faculty of Veterinary Medicine, University of Tehran, Tehran, Iran; 4 Department of Cardiology, Dr Shariatee training and research Hospital, Tehran University of Medical Sciences, Tehran, Iran; 5 Sleep Disorders Research Center, Kermanshah University of Medical Sciences, Kermanshah, Iran

**Keywords:** Coronary artery disease, Asprosin, Insulin resistance, Dyslipidemia, Adipokine

## Abstract

**Background:**

Coronary artery disease (CAD) is considered as a multi-faceted chronic inflammatory disease involving reduced blood supply to the myocardium as a result of accumulating lipids in the atrial walls. Visceral adiposity with disrupted release of adipokines play a key role in its pathogenesis. Asprosin is a newly identified fasting-induced glucogenic adipokine that has been related with metabolic disorders such as type II diabetes mellitus and polycystic ovary syndrome. The preset study sought to assess circulating asprosin in context of CAD.

**Methods:**

In this study, serum levels of asprosin were determined in 88 CAD patients and 88 non-CAD healthy controls. Serum IL-6, TNF-α, asprosin and adiponectin were assessed using ELISA kits.

Results: Serum asprosin was found to be higher in CAD patients when compared to non-CAD subjects (7.84 ± 2.08 versus 5.02 ± 1.29 μg/mL, *p* <  0.001). Similarly, serum TNF-α, and IL-6 elevated in CAD group significantly (*p* <  0.001). However, circulating adiponectin diminished in CAD group when compared with non-CAD subjects (*p* < 0.001). Moreover, serum asprosin levels directly correlated with BMI, FBG, HOMA-IR, TG and TC. Logistic regression analyses showed that asprosin levels were associated with increased risk of developing CAD (odds ratio: 3.01, 95% CI: 2.16, 4.20 and *p* < 0.001), after adjusting for potential confounders (age, sex and BMI).

**Conclusions:**

The present study findings suggested a possible relation of serum asprosin with the pathogenesis of CAD, in particular through insulin resistance and dyslipidemia.

## Introduction

Coronary artery disease (CAD), affects 1655 per 100,000 worldwide, and is expected to exceed 1845 by 2030. It is the main cause of death globally, with 9 million deaths annually [1]. Despite exceptional medical advancements, CAD continues to exert a global economic burden [2]. Pathologically, it is a chronic inflammatory disease state, with a concomitant accumulation of cholesterol and activation of both innate and adaptive immune responses, and eventually leading to atherosclerosis [3].

Obesity, central adiposity in particular, exhibits a complex relationship with cardiovascular diseases (CVD); primarily by influencing the pathogenesis and the severity of comorbidities such as hypertension and glucose intolerance, and secondarily by altering the structure and function of the myocardium [4, 5]. Inflammation is a key pathological mechanism in the pathogenesis of atherosclerosis. In first step, oxidation of low density lipoprotein (LDL) in the subendothelial space produces oxidized-LDL (Ox-LDL) that recruits monocytes from circulation. These immune cells differentiate to macrophages and they uptake Ox-LDL via their scavenger receptors. This condition leads to formation of foam cells and eventually plaque formation [6]. The bidirectional induction of inflammation by the accumulating lipids in the endothelium, tissue infiltration by macrophage, and excess adiposity results in the release of adipokines (resistin, leptin, tumor necrosis factor (TNF)-α, visfatin, retinol binding protein (RBP)-4) by the adipose tissue, culminating with insulin resistance, endothelial dysfunction, prothrombotic state and systemic inflammation [3].

Asprosin is recently discovered as a C-terminal cleavage product of profibrillin, mainly synthesized by white adipocytes, but might also be excreted by β-pancreatic cells [7]. It is expressed in tissues including, placenta in humans [8], and surface epithelial cells of stomach fundus, cortical distal tubule of the kidneys, and cardiomyocytes of the heart in rats [9]. Asprosin crosses the blood-brain barrier to induce the hypothalamic feeding circuits, while navigating hepatic glucose release via the signaling pathway of G-protein cyclic adenosine monophosphate (cAMP)/protein kinase A (PKA); thereby, stimulating appetite and influencing adiposity [7, 10]. Moreover, it is involved in ovarian follicular function [11, 12], insulin sensitivity [13], inflammation and apoptosis [14]. Asprosin has been implicated in neonatal progeroid syndrome [7], Marfan syndrome [15], malignant mesothelioma [16] and post-burn treatment [17]. Moreover, studies have shown relation of circulating asprosin with some metabolic disorders including diabetes mellitus [7, 18–22], obesity [10, 23], and polycystic ovary syndrome (PCOS) [20, 24]. On the one hand, pathologically elevated levels of asprosin were found in diabetic and obese individuals, and on the other hand its neutralization resulted in reduced food intake and lower glucose and insulin levels [7]. Current information suggests a promising role for asprosin as a therapeutic target in metabolic disorders.

While asprosin play a role in several CAD risk such as insulin resistance and inflammation, the levels of this asprosin in individuals diagnosed with CAD has not been determined. This study sought to determine levels of asprosin in CAD and non-CAD subjects, as well as its relation with metabolic parameters.

## Study population and methods

### Study design, settings, and participants

This case-control study was performed in accordance with the Declaration of Helsinki. The study approved by the ethical committee of Tehran University of Medical Sciences (ethical code: IR.TUMS.MEDICINE.REC.1398.678). All subjects signed a written informed consent.

This case-control study carried out on 88 CAD patients (CAD group) and 88 controls (non-CAD group), 40—75 years of age. Patients were the people who referred to angiography unit because of having the indicators of CAD from Jan 2020 to Dec 2020. The inclusion criteria for CAD group was a clear diagnosis by a cardiologist of having at least one coronary artery with 50% ≤ stenosis in the angiography [25]. CAD group was further subdivided into single-, double-, and triple-vessel disease subgroups based on the number of vessels that had at least 50% stenosis. Moreover, patients with unstable angina or myocardial infraction were excluded from the CAD group. On the other hand, non-CAD group included subjects with arterial stenosis less than 30%, according to angiographic image. Individuals suffering from diabetes mellitus chronic illness such as hepatic disease, renal disease, or stroke; inflammatory, tumor, allergic, or autoimmune diseases; or hematologic diseases; or receiving lipid lowering, insulin, or immunosuppressant agents were excluded from the study.

### Anthropometrics and biochemical measurements

Body mass index (BMI) was detrmined using a standard formula [weight (Kg)/ height^2^ (m^2^)]. Following a 15-min rest in setting position, systolic (SBP) and diastolic blood pressure (DBP) were measured using a standard sphygmomanometer. Then, after an overnight fasting (8–12 h.), five milliliters of venous blood were obtained. The circulating levels of fasting blood sugar (FBS), fasting insulin, total cholesterol (TC), triglycerides (TG), high-density lipoprotein cholesterol (HDL-C), LDL-C, creatinine (Cr), aspartate aminotransferase (AST) and alanine aminotransferase (ALT) were measured by available kits (ParsAzmoon, Tehran, Tehran, Iran). The homeostatic model assessment of insulin resistance (HOMA-IR) was determined by applying the following equation: [(FBG (mg/dL)] × [fasting blood insulin (μU/mL)] / 405 [26].

### Measuring adipokines and cytokines

Serum levels of TNF-α and inerleukin-6 (IL-6) (R&D system, Minneapolis, MN, USA) were determined using ELISA technique, the minimum detectable values were 1.6 and 0.7 pg/mL, respectively. Serum levels of asprosin were assessed using commercial ELISA kit (Aviscera Bioscience, Santa Clara, CA, USA). The intra- and inter-assay coefficients of variation (CV) of Asprosin were 6 and 8%, respectively. Adiponectin levels were determined using ELISA kit (Adipogen, South Korea) with intra- and inter-assay of 4.6 and 4.4%, receptively.

### Statistical analysis

IBM SPSS Statistics 20 (IBM SPSS, Chicago, IL, U.S.A.) was applied to perform statistical analyses. Categorical data are presented by frequency and percentage, and Chi-squared test was applied to compare between the groups. Continuous variables were presented as mean and standard deviation (SD), and were tested using student t-test and one-way ANOVA with Bonferroni post hoc test. Spearman correlation test was used to determine the correlation of serum asprosin levels with the continuous variables. Then, multiple linear regression carried out to investigate those associations. The association of asprosin with CAD risk was estimated using binary logistic regression. The confounders (age, sex, and BMI) were added to the model for adjustment. All tests were two-sided, and a *p*-value of less than 0.05 was considered statistically significant.

## Results

### Basic characteristics of the study population

The clinical data of the studied groups are outlined in Table 1. CAD group showed no statistical difference with the control group in terms of age, BMI, sex, SBP, DBP, and Cr. Regarding parameters of glucose metabolism, CAD group indicated elevated levels of insulin and HOMA-IR (*p* < 0.001), but not FBG, when compared to non-CAD group. The levels of TG elevated (*p* < 0.001) and HDL-C decreased in patient group compared to non-CAD subjects (*p* = 0.021), while TC and LDL-C indicated no considerable change between the groups. Finally, serum levels ALT and AST were found to be higher in CAD patients when compared to non-CAD group (*p* < 0.05).

**Table 1:** Clinical characteristics of the studied population

Variables	Non-CAD (***n*** = 88)	CAD (***n*** = 88)	***P***
**Age (year)**	**58.25 ± 8.46**	**59.19 ± 7.9**	**0.446**
**Sex [male]**	**63 (71.6%)**	**58 (65.9%)**	**0.258**
**BMI (kg/m2)**	**25.23 ± 3.52**	**25.65 ± 3.86**	**0.457**
**SBP (mmHg)**	**127.25 ± 16.38**	**130.61 ± 18.97**	**0.210**
**DBP (mmHg)**	**80.43 ± 11.22**	**83.22 ± 13.19**	**0.133**
**FBG (mg/dL)**	**93.49 ± 12.2**	**95.38 ± 11.98**	**0.303**
**Insulin (uU/mL)**	**4.07 ± 2.9**	**6.11 ± 4.03**	**< 0.001**
**HOMA-IR**	**0.96 ± 0.69**	**1.48 ± 1.018**	**< 0.001**
**TG (mg/dL)**	**115.9 ± 46.83**	**143.94 ± 55.06**	**< 0.001**
**TC (mg/dL)**	**165.52 ± 36.46**	**176.27 ± 46.39**	**0.089**
**LDL-C (mg/dL)**	**99.89 ± 29.74**	**106.85 ± 31.32**	**0.132**
**HDL-C (mg/dL)**	**44.28 ± 7.64**	**41.26 ± 9.49**	**0.021**
**Cr (mg/dL)**	**1.13 ± 0.17**	**1.13 ± 0.17**	**0.944**
**AST (U/L)**	**16.24 ± 4.62**	**18.5 ± 5.79**	**0.005**
**ALT (U/L)**	**16.5 ± 6.69**	**19 ± 7.27**	**0.019**

### Serum levels of adipokines and cytokines

**Fig. 1:**
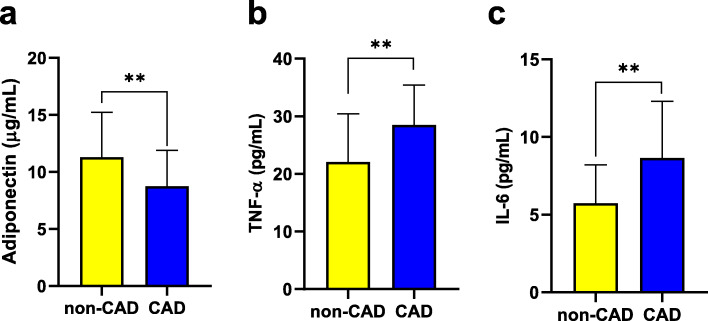
Circulating levels of adiponectin, TNF-α and IL-6. **a** Serum levels of adiponectin decreased in CAD group compared to controls. **b** Serum levels of TNF-α and **c** IL-6 elevated in CAD patients compared to non-CAD subjects

**Fig. 2:**
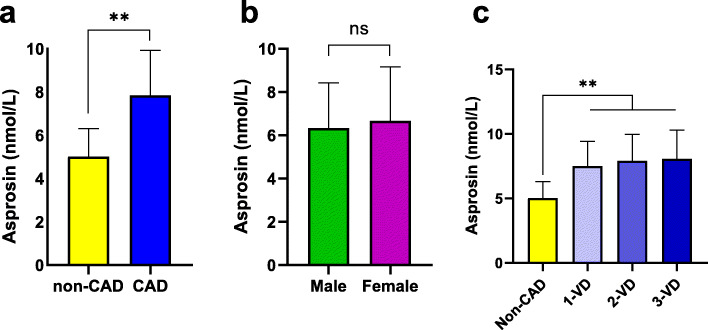
Serum levels of asprosin in according to disease status, sex and stenosed vessels. **a** Serum levels of asprosin increased in CAD compared to non-CAD group. **b** Asprosin serum levels indicated no significant change between male and female. **c** Asprosin elevated in all three subgroups of CAD patients compared to non-CAD group, while there were no considerable change between patients according to number stenosed vessels. VD, vessel disease

Adiponectin serum levels in CAD group (8.72 ± 3.15 μg/mL) were lower than controls (11.28 ± 3.95 μg/mL, *p* < 0.001) (Fig. 1a). While, serum levels of TNF-α (28.48 ± 6.93 vs. 22.09 ± 8.32 pg/mL) and IL-6 (8.64 ± 3.65 vs. 5.73 ± 2.46 pg/mL), were substantially higher in CAD group compared to control subjects (*p* < 0.001) (Fig. 1b and c). Similarly, levels of asprosin were elevated considerably in CAD patients (7.84 ± 2.08 nmol/L), when compared to controls (5.02 ± 1.29 nmol/L, *p* < 0.001) (Fig. 2a). In addition, serum asprosin were tested between male (6.33 ± 2.1 nmol/L) and female (6.66 ± 2.5 nmol/L) and the results showed no considerable change between the groups (Fig. 2b). Circulating asprosin were significantly lower in subjects with no stenosed vessels, i.e. non-CAD group (5.02 ± 1.29 nmol/L), when compared to those with stenosed vessels (*p* < 0.001). There were no considerable change in asprosin serum levels between 1-vessel disease (7.49 ± 1.92 nmol/L), 2-vessel disease (7.9 ± 2.06 nmol/L) and 3-vessel disease (8.07 ± 2.22 nmol/L) subgroups (Fig. 2c).

Moreover, the association of serum asprosin with CAD risk was tested via binary logistic regression analysis. A considerable association between asprosin levels and CAD was observed (odds ratio: 2.67, 95% confidence interval: 1.99—3.60 and *p* < 0.001). Adjustment for potential confounders (age, gender and BMI) did not affect the independent association between the two variables (OR: 3.01, 95% CI], *p* < 0.001) (Table 2).

**Table 2:** Odd ratio (OR) of CAD risk according to asprosin levels

Model	OR	95% confidence interval	*P* value
Crude	2.67	1.99, 3.60	< 0.001
Adjusted	3.01	2.16, 4.20	< 0.001

Adjustment was performed for age, gender and BMI

**Fig. 3:**
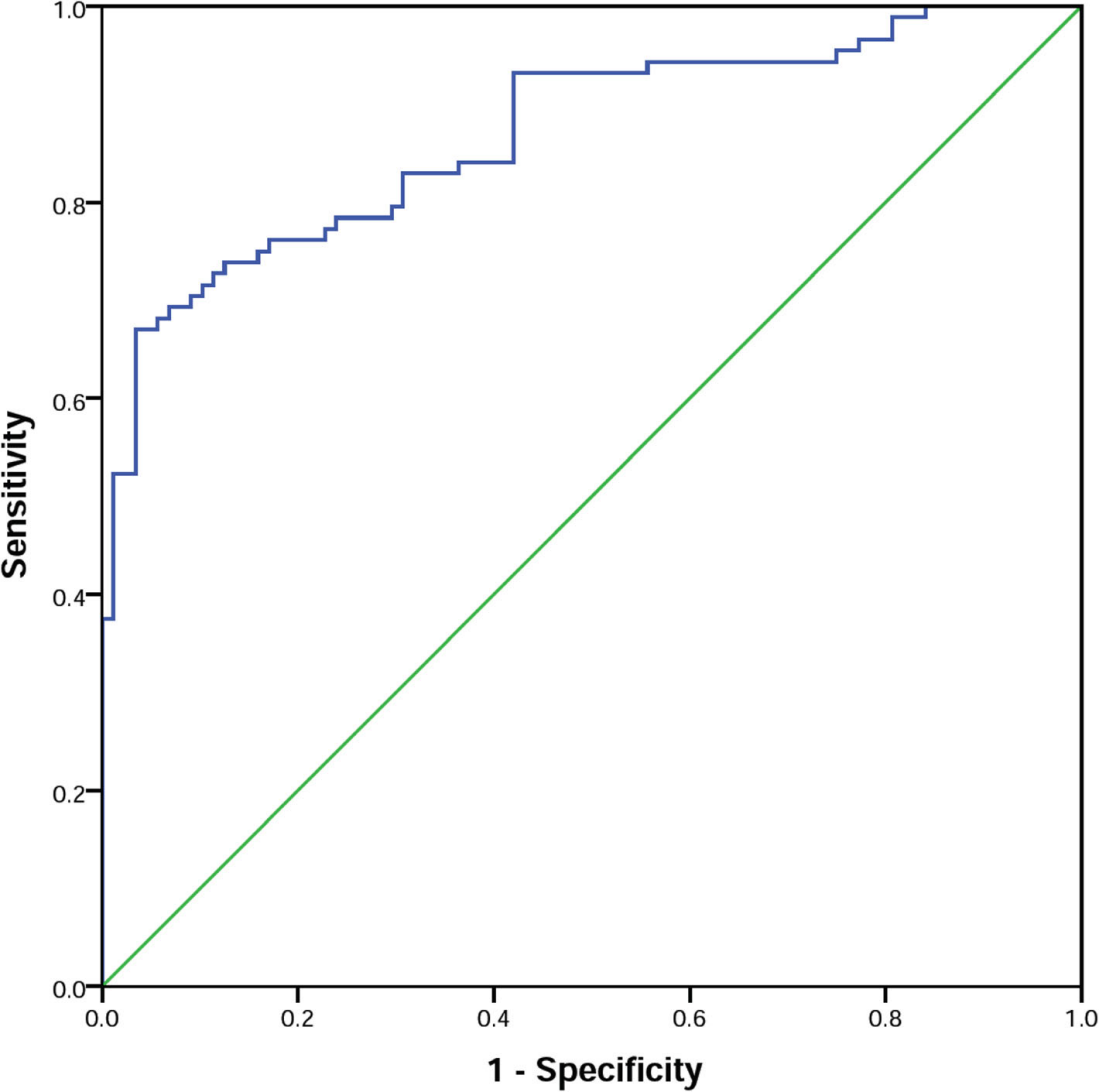
ROC curve for diagnostic ability of asprosin for distinguish between CAD and non-CAD status

Moreover, the diagnostic ability of asprosin was tested using receiver operating characteristic (ROC) curve analysis. The findings indicated that a cut-off value of 6.05 nmol/L had a good sensitivity (78.4%) and specificity (76.1%) to distinguish CAD from non-CAD (Area under curve [95% CI]: 0.870 [0.818—0.923], *p* < 0.001) (Fig. 3).

### Association of serum asprosin with clinical parameters

The results of correlation analyses of serum asprosin with other parameters are presented in Table 3. In non-CAD group, serum asprosin levels positively correlated with FBG, HOMA-IR, BMI, TG, and ALT. Multiple stepwise linear regression using correlated factors showed that FBG (B [95% CI]: 0.04 [0.019—0.061], *p* < 0.001) had the strongest association with asprosin in controls. On the other hand, in CAD group, serum asprosin positively correlated with BMI, FBG, HOMA-IR, TG, and TC. Moreover, multiple stepwise linear regression indicated that asprosin had the strongest association with FBG (B [95% CI]:0.04 [0.008—0.073] *p* = 0.016), BMI (B [95% CI]: 0.168 [0.066—0.269], *p* = 0.002) and TC (B [95% CI]: 0.014 [0.005—0.022], *p* = 0.002) in the CAD group.

**Table 3:** Pearson correlation of serum Asprosin levels with anthropometric and biochemical variables

Variables	Pearson Correlation (r)	
	No-CAD	CAD
**Age (year)**	0.017	−0.194
**BMI (kg/m2)**	0.248^*^	0.392**
**SBP (mmHg)**	0.158	−0.020
**DBP (mmHg)**	0.096	−0.022
**FBG (mg/dL)**	0.377^**^	0.252*
**Insulin (uU/mL)**	0.196	0.200
**HOMA-IR**	0.277^**^	0.246*
**TG (mg/dL)**	0.255^*^	0.245*
**TC (mg/dL)**	0.076	0.338**
**LDL-C (mg/dL)**	−0.032	0.180
**HDL-C (mg/dL)**	−0.189	0.045
**Cr (mg/dL)**	−0.125	−0.019
**AST (U/L)**	0.073	0.161
**ALT (U/L)**	0.271^*^	0.179
**TNF-α (pg/mL)**	0.126	0.053
**IL-6 (pg/mL)**	0.084	0.128
**Adiponectin (μg/mL)**	−0.178	−0.055

* *P* < 0.05, **P < 0.01

## Discussion

Asprosin has been recently identified as a fasting-induced glucogenic hormone [7]. There is little data on its association with CAD. The current study, found that circulating levels of asprosin are significantly elevated in CAD patients when compared to controls, for the first time. While there are no data on comparing asprosin between CAD patients and controls, Acara et al. found that asprosin levels significantly correlated with the severity of disease in unstable angina pectoris patients [27]. On other hand, in a five-year follow-up cohort study, Wen et al. found that high levels of asprosin were protective against adverse cardiac events in dilated cardiomyopathy patients [28]. Decreased asprosin were related with higher risk of worse clinical outcomes. The authors suggested that asprosin directly protects cardiomyocytes by improving mitochondrial respiration in response to hypoxia [28]. Moreover, it has been shown that other adipokines such as adiponectin, Von Willebrand factor and Lp(a) had relation with CAD [29–31].

Excess energy is stored in adipocytes as fat, which eventually leads to adiposity and disrupted levels of adipokines, resulting in insulin resistance and dyslipidemia, and eventually increasing the risk of developing adverse cardiovascular events [32–34]. Increased adiposity releases higher levels of asprosin, which in turn induces an increase in glucose output [35]. Ozcan et al. found that irisin administration induces an increase in the serum levels of asprosin in healthy male rats, but not in obese ones [36]. On the other hand, aerobic exercise training and bariatric surgery were successful at lowering asprosin levels in overweight/obese individuals [37–39]. In the current study, asprosin level positively correlated with BMI that was in accordance with the findings were seen in previous studies [18, 19]. However, the association of asprosin with adiposity is quite contradictory. For instance, individuals diagnosed with anorexia nervosa demonstrated elevated levels of asprosin [40]; while patients diagnosed with cancer-related anorexia had significantly lower levels of asprosin, positively correlating with body fat mass [41]. Moreover, a cohort study of 444 patients with PCOS and 156 controls found that circulating asprosin were independent of BMI in PCOS subjects [42]. On the contrary, Li et al. found that overweight subjects showed high levels of asprosin regardless of their metabolic disease (diabetic or PCOS) [20]. It also showed a positive association with waist circumference, waist-to-hip ratio and BMI in diabetic individuals [18, 19]. Moreover, inconsistent results were demonstrated in children with obesity. Long et al. found that children with obesity had lower levels of asprosin when compared to normal weight controls [43]. However, Wang et al. showed that obese children exhibited high levels of asprosin when compared to controls, associating with insulin resistance [23]. A recent study in Turkish population found similar results [44]. Intriguingly, there is dramatic discrepancy in circulating levels of asprosin among studies. These conflicting results might be due to difference in disease status, population, or ELISA kit used. Nevertheless, once again asprosin is seen as a complex energy regulator. This infers a need for further studies with the use of more accurate estimators of adiposity than BMI.

The results showed that circulating asprosin levels positively correlated with TG in non-CAD group and with TC in CAD group. Similar results were demonstrated in previous studies. Asprosin levels associated with TG and TC/HDL-C ratio in diabetic individuals [18, 19, 45], and LDL-C, apolipoprotein (APO) B, and APO E in polycystic ovarian syndrome (PCOS) [20]. There are several line of evidence for the relation of asprosin with metabolism of lipoprotein, and this flinging of the preset study provide another evidence for this relationship in CAD patients.

Insulin resistance, rather than glycemic control, is an independent predictor of a cardiovascular event among non-diabetic individuals [46, 47]. Asprosin has been identified as a ‘fasting-induced hormone’ that directly stimulates hepatic glucose output by binding to olfactory receptor Olfr734 on hepatocyte surface and upregulating hepatic cAMP levels [7, 48]. Lee et al. found that, under hyperlipidemic conditions, pancreatic β-cells release asprosin, elucidating inflammation, cellular dysfunction and apoptosis via toll-like receptor-4 (TLR-4) / c-Jun N-terminal kinase (JNK) phosphorylation, that eventually leading to insulin resistance [14]. Recently, Wang et al. demonstrated that asprosin induces apoptosis of β-cells by inhibiting their autophagy via adenosine monophosphate-activated protein kinase (AMPK) and mammalian target of rapamycin (mTOR) signaling pathway [49]. Moreover, in C2C12 myocytes, asprosin treatment augmented insulin resistance by impairing insulin receptor substrate (IRS)-1 and Akt phosphorylation [13]. On the other hand, immunologic or genetic loss of function resulted in a significant decrement in insulin and glucose levels, providing a protective mechanism against hyperinsulinemia and insulin resistance [7]. In the current study, serum levels of asprosin positively correlated with FBG and HOMA-IR in both groups, but not enough to create an independent association. This might be due to less pronounced insulin resistance in the CAD participants. Future investigation with an additional T2DM and CAD-T2DM groups might unravel better insight. Nevertheless, several studies have found that asprosin levels are pathologically elevated in individuals with insulin resistance, negatively correlated with FBG, HbA1c, and HOMA-IR [7, 18–20, 45]. Wang et al. found that it might contribute to β-cell dysfunction and glucose intolerance in patients with T2DM [19]. Recently, Gozel et al. reported that both serum and saliva levels of asprosin are significantly higher in newly diagnosed T2DM when compared to normoglycemic controls [50]. Individuals diagnosed with other metabolic disorders such as gestational diabetes, PCOS and non-alcoholic fatty liver disease also showed significantly higher asprosin concentration compared to their normal counterparts, showing a positive correlation with insulin resistance [22, 24, 51–53].

### Study strength and limitations

The present study evaluated asprosin in angiography-confirmed CAD patients in comparison to control for the first time. There are few limitations. First, the methodological approach limits a causal relation between the two variables. Second, having additional T2DM and CAD-T2DM groups might have been helpful on clearing the association of asprosin with CAD on the basis of insulin resistance. Third, assessing EAT of the participants would have been insightful on the relationship of asprosin released by this type of adipocytes with CAD pathogenesis.

## Conclusion

In conclusion, asprosin levels are pathologically elevated in CAD patients, correlated with adiposity, dyslipidemia and insulin resistance, that suggested a possible relation of asprosin with pathological mechanism of CAD, can be considered for targeting insulin resistance and dyslipidemia in these patients. Moreover, it might considered as a possible biomarker. However, the mechanism behind this required to be investigated.

## Data Availability

The data that support the findings of this study are available on request from the corresponding author.
